# Case report: Sodium-glucose cotransporter 2 inhibitors induce left ventricular reverse remodeling in anthracycline-related cardiac dysfunction—a case series

**DOI:** 10.3389/fcvm.2023.1250185

**Published:** 2023-08-22

**Authors:** Francesco Giangiacomi, Andrea Faggiano, Daniela Cardinale, Francesca Gaia Rossi, Alberto Pollina, Elisa Gherbesi, Eleonora Gnan, Stefano Carugo, Marco Vicenzi

**Affiliations:** ^1^ Department of Cardio-Thoracic-Vascular Diseases, Foundation IRCCS Ca’ Granda Ospedale Maggiore Policlinico, Milan, Italy; ^2^ Dyspnea Lab, Department of Clinical Sciences and Community Health, University of Milan, Milan, Italy; ^3^ Cardioncology Unit, European Institute of Oncology, IRCCS, Milan, Italy; ^4^ Haematology Unit, Foundation IRCCS Ca’ Granda Ospedale Maggiore Policlinico, Milan, Italy

**Keywords:** sodium-glucose cotransporter 2 inhibitors, anthracycline-related cardiac dysfunction, left ventricular remodeling, cardiotoxicity, heart faillure

## Abstract

**Purpose:**

To describe the efficacy and safety of sodium-glucose cotransporter 2 inhibitors as a specific treatment for anthracycline-related cardiac dysfunction in a small real-world population.

**Methods:**

Seven patients with anthracycline-related cardiac dysfunction were clinically and echocardiographically evaluated before and after the introduction of sodium-glucose cotransporter 2 inhibitors.

**Results:**

After a median period of 24 weeks with uninterrupted sodium-glucose cotransporter 2 inhibitors treatment, a significant clinical improvement was observed with at least one New York Heart Association Functional Class (NHYA FC) improvement in all patients (median NYHA FC: I vs. III, *p* < 0.010). A noteworthy left ventricular reserve remodeling (median left ventricular end diastolic volume indexed: 53 vs. 82.5 ml/m^2^, *p* = 0.018; median left ventricular ejection fraction: 50% vs. 40%, *p* = 0.17) was also observed. Sodium-glucose cotransporter 2 inhibitors therapy was well tolerated by every patients; no cases of discontinuation or relevant side effects were observed.

**Conclusion:**

Sodium-glucose cotransporter 2 inhibitors induce a significant clinical improvement and left ventricular reserve remodeling in patients affected by anthracycline-related cardiac dysfunction.

## Highlights

• Sodium-glucose cotransporter 2 inhibitors induce a significant clinical improvement in patients affected by anthracycline-related cardiac dysfunction.
• Sodium-glucose cotransporter 2 inhibitors induce a significant increase in left ventricle ejection fraction and reduction in indexed left ventricle end-diastolic volumes.

## Introduction

Cardiovascular disease and cancer are the main leading causes of death in developed countries. While advancements in oncological treatment have led to improved survival rates, they have also resulted in an increase in cardiac side effects. Anthracyclines are effective and widely used oncological treatments, although they are associated with the risk of severe myocardial damage which can often necessitate the discontinuation of chemotherapy. The European Society of Cardiology (ESC) has recently published the first edition of guidelines on cardio-oncology, defining anthracycline-related cardiac dysfunction (ARCD) as a form of heart failure (HF) characterized by left ventricle (LV) remodeling and mild-to-severe impairment of ejection fraction (1). Currently, no specific treatment is available, and all efforts are directed towards preventing damage and its progression. The ESC Guidelines on cardio-oncology recommend treating ARCD following the indications of the ESC Guidelines on HF. To highlight the potential role of sodium-glucose cotransporter 2 inhibitors (SGLT2-i) in preventing ARCD, our research team recently conducted a meta-analysis showing promising results on mouse models (2). Furthermore, two retrospective analysis of patients with diabetes and cancer provided preliminary evidence of the protective role of SGLT2-i in humans (3, 4). Given the potential cardioprotective effects of SGLT2-i, we investigated their efficacy in treating ARCD, as no evidence on this issue had been published. Here, we present a case series of consecutive patients diagnosed with ARCD who were given SGLT2-i in addition to guideline-directed medical treatment (GDMT).

## Case description

We collected seven cases of patients with ARCD and symptomatic HF with reduced ejection fraction (HFrEF). They had all been exposed to a high dose of anthracycline for treating different types of cancer. No other cause of HF was found, and coronary angiography was negative in all cases. All the patients had clinical and echocardiographic assessments in three different time periods: before chemotherapy (T0), after chemotherapy when ARCD was diagnosed (T1), and after SGLT2-i therapy (T2). New York Heart Association functional class (NYHA FC), two-dimension LV ejection fraction (LVEF), LV end-diastolic and volume indexed (LVEDVi), were considered to describe the clinical status and LV remodeling. We compared T0 vs. T1, T1 vs. T2, and T2 vs. T0.

The non-parametric statistical Wilcoxon Signed Rank Test was adopted. The significance level (*α*) was considered at a two-tailed probability level of significance of 95% (*p* < 0.05). The Chi2 test through Person's analysis was used to test NYHA functional class changes. The statistical analysis was carried out using SPSS version 28.0.1.0.

The demographic, clinical, and echocardiographic characteristics are reported in Table 1. Among the seven patients, six were female, and the median age was 55 years. All patients underwent chemotherapy with a high cumulative anthracycline dose (>250 mg/m^2^ of doxorubicin or equivalent). All the patients had a NYHA FC I before completing the chemotherapy cycles, and none had previous cardiovascular disease or diabetes. All patients had normal echocardiographic morpho-functional parameters before chemotherapy, and none had more than mild valvular disease.

**Table 1:** Demographic, clinical and echocardiographic morpho-functional characteristics.

Variable	Patient 1	Patient 2	Patient 3	Patient 4	Patient 5	Patient 6	Patient 7
Age at the beginning of CT, years	65	55	55	58	42	54	52
Sex	Female	Female	Female	Male	Female	Female	Female
Cancer	Mantle cell lymphoma	Mantle cell lymphoma	Follicular lymphoma	Hodkin's lymphoma	Breast cancer	Follicular lymphoma	B-cell lymphoma
Chemotherapy	HCDA	HCDA	HCDA	HCDA	HCDA	HCDA	HCDA
Time from CT to cardiotoxity, months	8	120	130	10	36	6	24
HF treatment after CT	BB, ARNI, MRA, furosemide	BB, ACE-I, furosemide	ARNI, ivabradine	BB, ACE-I	BB, ARNI, furosemide	BB, ACE-I, MRA	BB, ACE-I, MRA
Optimized and stable HF treatment duration prior SGLT2i, weeks	56	24	48	36	24	52	36
SGLT2i, type (dosage)	Empagliflozin (10 mg)	Dapaglifozin (10 mg)	Dapagliflozin (10 mg)	Dapaglifozin (10 mg)	Dapaglifozin (10 mg)	Dapaglifozin (10 mg)	Dapaglifozin (10 mg)
Follow-up duration after SGLT2i, weeks	24	44	20	24	36	36	12
Baseline							
NYHA class	I	I	I	I	I	I	I
LVEDVi, ml/m^2^	44.3	46.8	45.3	51.2	58.5	66.6	36.8
LVEF, %	65	58	61	66	60	60	62
After chemiotherapy, with HF therapy but without SGLT2-i							
NYHA class	III	III	III	II	III	II	II
LVEDVi, ml/m^2^	95.8	93.6	73.3	82.5	71.9	88	51.5
LVEF, %	20	36	40	40	26	40	40
After SGLT2-i							
NYHA class	II	II	I	I	I	I	I
LVEDVi, ml/m^2^	47.9	68.2	46.6	53	60.2	70.8	26.4
LVEF, %	37	46	50	50	54	44	51

CT, chemotherapy; HCDA, high cumulative dose of anthracyclines (>250 mg/m^2^ of doxorubicin or equivalent); BB, beta-blocker; ARNI, angiotensin receptor-neprilysin inhibitor; ACE-I, angiotensin-converting enzyme inhibitor; MRA, mineralocorticoid receptor antagonist; SGLT2i, sodium-glucose cotransporter 2 inhibitors; LV, left ventricle; EDVi, end-diastolic volume indexed; EF, ejection fraction; NYHA, New York Heart Association.

Since ARCD was diagnosed, all parameters showed a significant statistical difference from T0 to T1: patients presented with remarkable LV remodeling (median LVEDVi 46.8 vs. 82.5 ml/m^2^, *p* = 0.018), significant reduction in LVEF (median LVEF 61% vs. 40%, *p* = 0.018), and a relevant degradation of symptoms (median NYHA FC I vs. III, *p* < 0.001). At T1, all patients had been treated with at least two HF drugs for at least 6 months, the most common combination being a beta-blocker and angiotensin-converting enzyme inhibitor (ACE-I) or angiotensin receptor-neprilysin inhibitor (ARNI). Symptomatic hypotension was the main limitation to the simultaneous administration of GDMT for HFrEF. If indicated, cardiac resynchronization therapy (CRT) was implanted, and SGLT2-i initiation occurred at least 6 months after the implantation.

Of the seven patients, six were given dapagliflozin 10 mg and one empagliflozin 10 mg daily. After a median period of 24 weeks with uninterrupted SGLT2-i treatment, significant clinical improvement was observed at T2 compared to T1 with at least one NYHA FC improvement in all patients (median NYHA FC I vs. III, *p* < 0.010). We also observed noteworthy LV reserve remodeling (median LVEDVi 53 vs. 82.5 ml/m^2^, *p* = 0.018; median LVEF 50% vs. 40%, *p* = 0.17).

**Figure 1:**
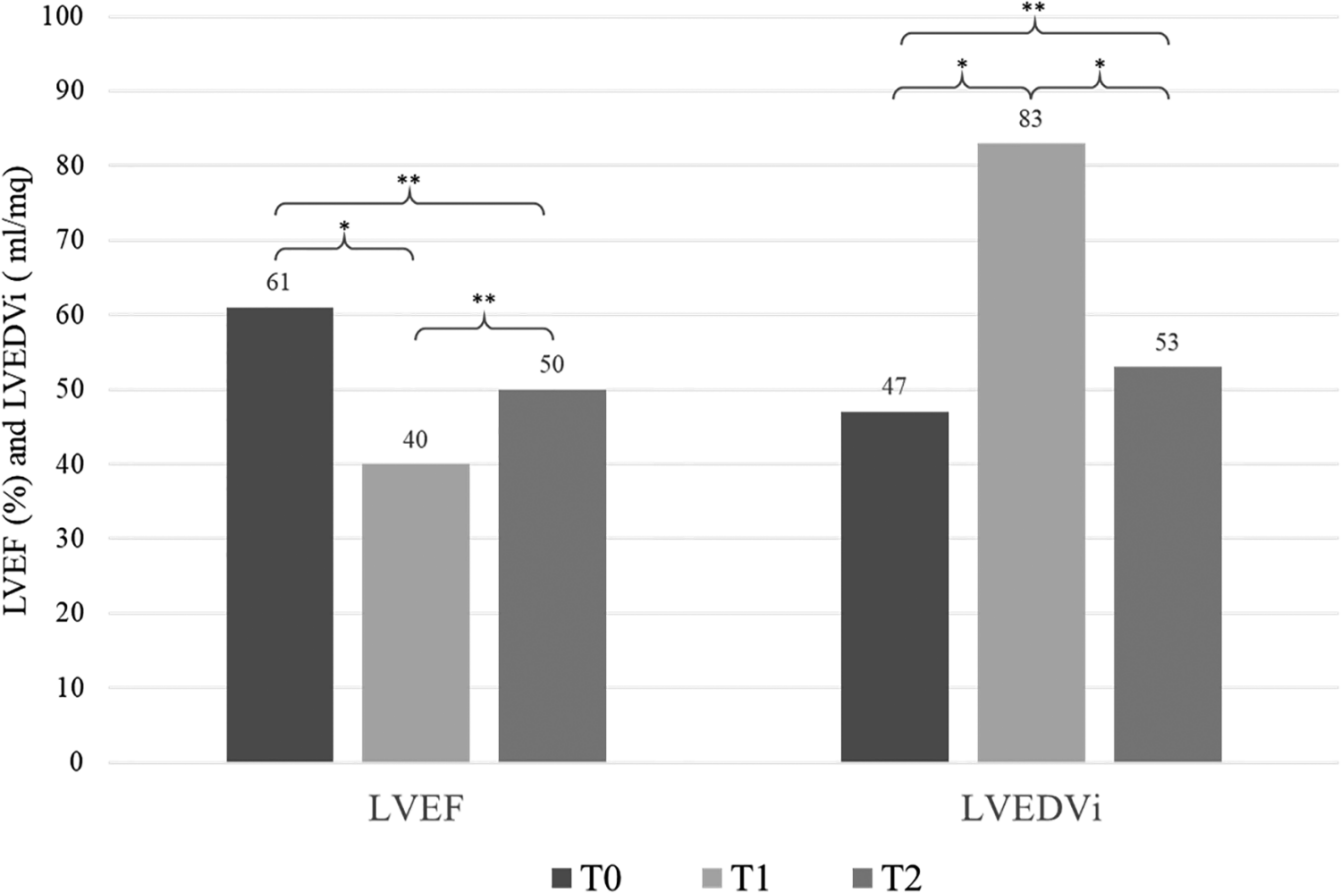
Echocardiographic variations over time (T0, T1, T2). T0 = pre-chemoteraphy, T1 = after chemotherapy when ARCD was diagnosed, T2 = after SGLT2-i therapy. LVEF, left ventricular ejection fraction; LVEDVi, left ventricular end diastolic volume indexed. *, *p* < 0.05; **, not significant.

No significant difference in clinical parameters was found when comparing T2 vs. T0, except for LVESV (median value 27.7 vs. 17.7 ml/m^2^, *p* = 0.028) (Figure 1).

SGLT2-i therapy was well tolerated by every patients; no cases of discontinuation or relevant side effects were observed.

We report below the two most significant cases:

### Case 1

Patient 1 was diagnosed with follicular lymphoma and treated with a cumulative doxorubicin equivalent dose >250 mg/m^2^. The echocardiographic follow-up showed normal LV volume and preserved LVEF, and the patient remained asymptomatic for 10 years following the end of chemotherapy. However, the patient showed severe dyspnea and LV systolic dysfunction. Cardiac magnetic resonance (CMR) revealed an initial dilatation of the LV (LVEDVi 106 ml/m^2^), a significant reduction in LVEF (34%), and a small LGE stria in the interventricular septum. Coronary angiography yielded negative results. Due to persistent symptoms and severe reduction in LVEF under GDMT, a CRT-D was implanted. One year after the implantation, clinical picture was unchanged. At this point, SGLT2-i was added to previous therapy [ARNI, mineralocorticoid receptor antagonist (MRA), and ivabradine]. After 6 months, LVEDVi and LVEF normalized (respectively 46.6 ml/m^2^ and 50%) and NYHA FC improved to I.

### Case 2

Patient 2 was diagnosed with Hodgkin's lymphoma and underwent chemotherapy with a cumulative doxorubicin equivalent dose >250 mg/m^2^. The echocardiography at baseline showed normal LV volumes and LVEF >60%. However, 1 year after the end of chemotherapy, the LV was mildly dilated (LVEDVi 82 ml/m^2^), and LVEF had decreased to 45%. The patient was started on treatment with bisoprolol and ramipril. Three months later, a further decrease in LVEF to 40% was observed through echocardiography. Therefore, CMR was performed, which revealed LV dilatation (105 ml/m^2^), diffuse hypokinesis, and a significant reduction in LVEF (37%). The patient also reported dyspnea during moderate physical activity (NYHA FC II). Coronary angiography was negative. At this time, SGLT2-i was added. At the follow-up 6 months later, the patient was asymptomatic (NYHA class I), and a recovery in LV volumes and contractility was observed (LVEDVi 46 ml/m^2^ and LVEF 50% at echocardiography).

## Discussion

To our knowledge, this is the first case series that demonstrates the benefits of SGLT2-i in ARCD. All patients showed a significant improvement in symptoms and echocardiography assessment after adding SGLT2-i to their HF GDMT. Additionally, we observed that SGLT2-i was well-tolerated and safe, with no cases of discontinuation or recorded side effects.

Despite none of our patients having a diabetes diagnosis, our findings are consistent with a recent retrospective study by Gongora et al. In their study, SGLT2-i were found to be effective in preserving LV function and reducing the incidence of cardiac events among patients with diabetes who were treated with anthracyclines. It is worth noting that no cases of ARCD were observed in the group treated with SGLT2-i (3). Our data suggest that SGLT2-i not only has a preventive role in patients at risk for ARCD but can also induce reverse remodeling. Although we cannot exclude the possibility of synergistic mechanisms with other concomitant drugs, we can speculate that SGLT2-i has pharmacodynamic characteristics that target the pathophysiology of ARCD.

Indeed, our research group recently reviewed basic science data supporting the potential role of SGLT2-i in preventing ARCD. Through a meta-analysis of 8 studies conducted on mouse models we showed that LVEF after chemotherapy with anthracyclines was significantly higher in rats preventively treated with SGLT2i (2). Additionally, SGLT2i have been shown to decrease plasma levels of cardiac troponin, brain natriuretic peptide, tumor necrosis factor-alfa, and fibroblast growing factor-2 in mouse models treated with anthracyclines. Myocardial fibrosis, intracellular radical oxygen species (ROS) formation, and lipid peroxidation were also lower in rats treated with SGLT2-inhibitors and anthracycline than in rats treated with only anthracycline (5, 6). Taken together, this evidence supports the hypothesis that SGLT2i may play a key role in the prevention and management of ARCD.

The pathophysiological mechanisms of ARCD are complex and include oxidative stress, intracellular ROS formation, lipid peroxidation, mitochondrial dysfunction, intracellular calcium dysregulation and microenvironmental cardiac inflammation (7). Therefore, the effect of SGLT2-i on cardiac metabolism, mitochondrial function, intracellular calcium homeostasis and inflammation may account for a specific and targeted cardioprotective effect. Indeed, SGLT2-i induces a shift of cardiac metabolism toward oxidation of fatty acids and consumption of ketone bodies, while reducing glucose utilization. Ketone bodies exert an anti-inflammatory effect and their use have been shown to improve cardiac function and remodeling in a canine model of cardiomyopathy and in nondiabetic subjects with HF. This substrate shift is also associated with an increase in myocardial adenosine triphosphate (ATP) content (8). Moreover, SGLT2-i are supposed to improve mitochondrial function and autophagy, thus reducing ROS formation. Gliflozins also directly inhibits Na^+^/Hydrogen exchanger 1, consequently reducing cytoplasmatic sodium and calcium concentrations, and increasing mitochondrial calcium concentration. This effect on calcium homeostasis is likely to improve myocardial contractility and reduce oxidative stress. Finally, SGLT2-i reduce inflammation, which plays a crucial role in the pathogenesis of ARCD. This effect is mediated by an increased activation of 5' adenosine monophosphate-activated protein kinase and a reduction in pro-inflammatory cytokines (9).

## Patient perspective and conclusions

Currently, evidence regarding the management of ARCD is scarce, and no trial on the use of SGLT2-i in this specific context has been published. Indeed, in previous large studies demonstrating the benefit of SGLT2-i, patients with active cancer were systematically excluded. Some promising data are expected from an ongoing randomized clinical trial (EMPACT trial, NCT05271162), which is investigating the efficacy of SGLT2-i in preventing ARCD.

At present, the ESC guidelines on cardio-oncology recommend treating patients with symptomatic ARCD and asymptomatic moderate or severe ARCD as indicated by HF guidelines. Therefore, the concomitant use and up-titration at the maximal tolerated dose of a beta-blocker, an ACE-I/angiotensin II receptor blocker (ARB) or ARNI, a MRA and a SGLT2-I is suggested (1). However, in clinical practice, it may be challenging to manage the treatment with all recommended HF drugs in patients with ARCD because of clinical frailty. Indeed, as shown in this case series, only one patient of seven was treated with four pharmacological classes (patient 1 in Table 1), and no specific indications are available on how to start and up-titrate treatments. The early diagnosis and initiation of targeted treatment are crucial to obtain a recovery of LV function in HF as well as in ARCD and beta-blockers and ACE-Is have been effective in improving prognosis (10). Our observation suggests that SGLT2-i should be considered as front-line treatment in ARCD together with the rest of the validated strategies. SGLT2-i showed a crucial role to induce reverse remodeling and restore cardiac function in ARCD. Despite obtaining a before-after evaluation someway allows each patient to serve as their own control, the main limitation of our study is precisely the lack of a control group. Prospective study and controlled clinical trials are needed to address properly this issue and obtain definitive results.

## Data Availability

The raw data supporting the conclusions of this article will be made available by the authors, without undue reservation.
